# Neurotrophic Requirements of Human Motor Neurons Defined Using Amplified and Purified Stem Cell-Derived Cultures

**DOI:** 10.1371/journal.pone.0110324

**Published:** 2014-10-22

**Authors:** Nuno Jorge Lamas, Bethany Johnson-Kerner, Laurent Roybon, Yoon A. Kim, Alejandro Garcia-Diaz, Hynek Wichterle, Christopher E. Henderson

**Affiliations:** 1 Project A.L.S./Jenifer Estess Laboratory for Stem Cell Research, New York, New York, United States of America; 2 Center for Motor Neuron Biology and Disease, Columbia University Medical Center, New York, New York, United States of America; 3 Department of Rehabilitation and Regenerative Medicine, Columbia University Medical Center, New York, New York, United States of America; 4 Department of Pathology and Cell Biology, Columbia University Medical Center, New York, New York, United States of America; 5 Department of Neurology, Columbia University Medical Center, New York, New York, United States of America; 6 Department of Neuroscience, Columbia University Medical Center, New York, New York, United States of America; 7 Columbia Stem Cell Initiative, Columbia University Medical Center, New York, New York, United States of America; 8 Columbia Translational Neuroscience Initiative, Columbia University Medical Center, New York, New York, United States of America; 9 Life and Health Sciences Research Institute, School of Health Sciences, University of Minho, Braga, Minho, Portugal; 10 ICVS/3B’s-PT Government Associate Laboratory, Braga/Guimarães, Minho, Portugal; University of Freiburg, Germany

## Abstract

Human motor neurons derived from embryonic and induced pluripotent stem cells (hESCs and hiPSCs) are a potentially important tool for studying motor neuron survival and pathological cell death. However, their basic survival requirements remain poorly characterized. Here, we sought to optimize a robust survival assay and characterize their response to different neurotrophic factors. First, to increase motor neuron yield, we screened a small-molecule collection and found that the Rho-associated kinase (ROCK) inhibitor Y-27632 enhances motor neuron progenitor proliferation up to 4-fold in hESC and hiPSC cultures. Next, we FACS-purified motor neurons expressing the Hb9::GFP reporter from Y-27632-amplified embryoid bodies and cultured them in the presence of mitotic inhibitors to eliminate dividing progenitors. Survival of these purified motor neurons in the absence of any other cell type was strongly dependent on neurotrophic support. GDNF, BDNF and CNTF all showed potent survival effects (EC_50_ 1–2 pM). The number of surviving motor neurons was further enhanced in the presence of forskolin and IBMX, agents that increase endogenous cAMP levels. As a demonstration of the ability of the assay to detect novel neurotrophic agents, Y-27632 itself was found to support human motor neuron survival. Thus, purified human stem cell-derived motor neurons show survival requirements similar to those of primary rodent motor neurons and can be used for rigorous cell-based screening.

## Introduction

*In vitro* differentiation of specific cell types from human pluripotent stem cells (hPSCs) allows for molecular and functional analysis of cells that are otherwise inaccessible. This holds special promise in neurodegenerative diseases such as amyotrophic lateral sclerosis (ALS), where ethical and technical constraints prevent access to human spinal motor neurons [1]. Using protocols based on normal developmental pathways, it has proven possible to generate spinal motor neurons from both mouse and human embryonic stem cells (ESCs) [2]–[6]. These are an important source of new mechanistic insights into the developmental requirements of wildtype motor neurons in both species. Moreover, successful specification of motor neurons from human induced pluripotent stem cells (hiPSCs) has opened novel avenues for mechanistic analysis of neuronal cell death and drug testing in motor neuron disease models [1], [4]–[8]. Yet our knowledge of the survival requirements of human motor neurons remains limited.

Cultured motor neurons from rodent embryos served as the basis for identification of the neurotrophic factors responsible for keeping motor neurons alive during development [9]–[11] and the same factors significantly retard motor neuron death in animal models of ALS [12]. In parallel, motor neurons cultured from mouse models of ALS shed light on the mechanisms underlying neurodegeneration [13]. All these discoveries required the purification of motor neurons from the complex environment of the spinal cord. This approach allowed for identification of factors that act directly on motor neurons, significantly facilitated direct quantification of motor neuron survival, and opened the door to biochemical studies that would not have been possible in mixed cultures. Although this might be considered a reductionist approach, conclusions about both survival factors and cell death mechanisms were subsequently validated *in vivo* [14]–[21], demonstrating that the advantages of motor neuron purification outweigh concerns about the artificial nature of the assay. It is therefore important to extend such approaches to human motor neurons. However, standard protocols for hPSC differentiation generate mixed populations of spinal neurons of which motor neurons constitute a minority, and to date survival of purified motor neurons has necessitated generally co-culture with other cell types [22]–[25]. There is consequently a need for a robust survival assay based on purified human motor neurons.

Another challenge is that absolute numbers of motor neurons generated from hESC/hiPSCs by standard procedures are relatively low. During embryonic development in rodents, motor neurons are produced from a short-lived pool of committed ventral spinal progenitors expressing OLIG2, which are rapidly exhausted or converted to oligodendroglial progenitors [26], [27]. However, in contrast to mouse motor neurons, which are produced during a brief period between embryonic days 9 and 12, the period of human motor neuron generation spans approximately twenty days [28], [29]. This raises the possibility that agents that enhance proliferation of motor neuron progenitors might be used to increase the yield of human motor neurons in culture.

Here we have developed techniques that allow us both to amplify stem cell-derived motor neurons and to perform survival assays in the absence of other cell types. We first report that there is indeed significant ongoing motor neuron generation in cultures of differentiated hESCs. To exploit this so as to increase yield, we therefore screened for compounds that increase the number of motor neurons when applied over this period. We report that the ROCK inhibitor Y-27632 stimulates the proliferation of OLIG2-expressing progenitors, and increases the yield of motor neurons up to four-fold. Using amplified motor neurons from the Hb9::GFP hESC line, we next defined conditions for a robust survival assay using FACS-sorted motor neurons, and used it to demonstrate potent activity for three known neurotrophic factors as well as Y-27632 itself. These approaches should be of general interest for the preparation of human motor neurons on a large scale and for functional and biochemical studies of molecular processes controlling motor neuron genesis, survival and degeneration.

## Results

### Ongoing motor neuron generation in cultures of differentiated hESCs

**Figure 1:**
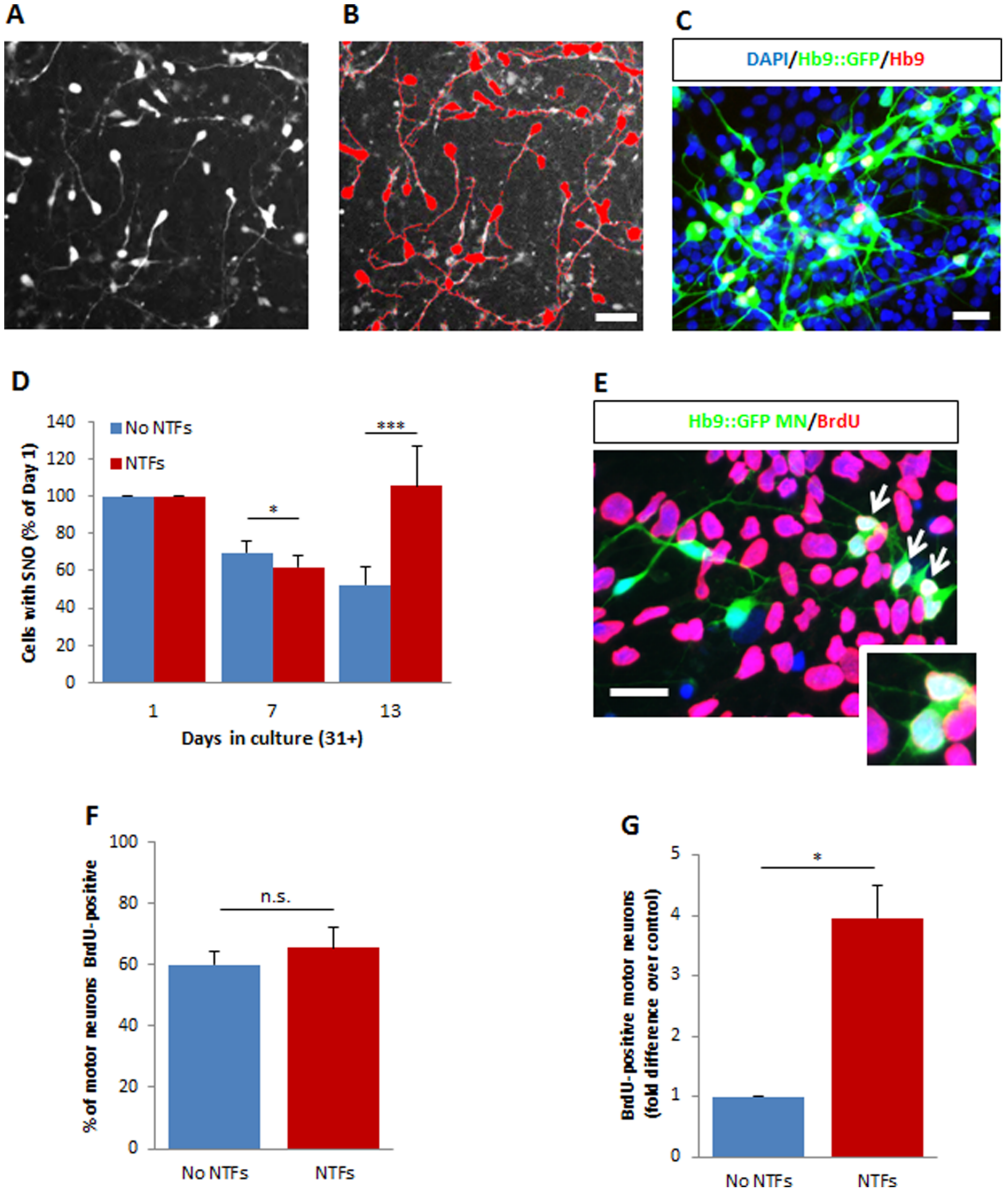
Ongoing birth of motor neurons in hESC-derived cultures is stimulated by neurotrophic factors. (A) Live fluorescent human motor neurons derived from the Hb9::GFP reporter line at day 31+13 after growth with a cocktail of neurotrophic factors (NTFs). (B) Automated quantification of fluorescent cells with significant neurite outgrowth (SNO) using the Neurite Outgrowth module of MetaMorph software; cells counted are identified with a red overlay. Motor neurons were considered to have significant neurite outgrowth when their overall neurite length exceeded 75 µm (scale bar). (C) Representative image of immunostained Hb9::GFP hESC-motor neuron cultures at day 31+13 after growth with a cocktail of neurotrophic factors (NTFs). Scale bar = 50 µM. (D) Number of cells with significant neurite outgrowth (SNO) when grown with (red bars) or without (blue bars) neurotrophic factors, expressed as a percentage of numbers at day 31+1. The increase in motor neuron numbers after day 31+7 in NTF-supplemented cultures suggests ongoing neurogenesis. Surviving fluorescent GFP-positive motor neurons with SNO shown as mean ± s.e.m., *n*>5 (t-test, ***p<0.001, *p<0.05). (E) BrdU-positive Hb9::GFP-positive motor neurons (arrows) at day 31+15 confirming the presence of newborn human motor neurons in culture. Scale bar = 50 µM. (F) The percentage of Hb9::GFP-positive motor neurons that were BrdU-positive at day 31+15 is not changed by NTFs but (G) total numbers of BrdU-positive motor neurons are increased with NTFs. Bars indicate mean ± s.e.m., *n* = 3 (t-test, *p<0.05; n.s.  =  not significant).

To determine whether hESCs differentiated *in vitro* to a mixed spinal cord identity exhibit prolonged motor neurogenesis as in the fetal human spinal cord, we first examined changes in numbers of hESC-derived motor neurons (hESC-MNs) in mixed spinal cultures over a 15-day period using an hESC reporter line that expresses green fluorescent protein (GFP) under the control of the motor neuron-specific murine homeobox gene 9 (Hb9) promoter [23]. We and others previously showed using a range of other markers and functional assays that GFP-positive neurons generated from this line possess many properties of postmitotic motor neurons [6], [23], [30]. Motor neurons were differentiated from hESCs using a standard protocol involving exposure of embryoid bodies (EBs) to retinoic acid (RA) and recombinant sonic hedgehog protein (SHH) (see *Methods*) [4], [6]. After 31 days, EBs were dissociated and cryopreserved to allow multiple experiments to be performed on identical aliquots; however, similar data were obtained using fresh, unfrozen cells (not shown). Cell suspensions were thawed and plated in 96-well plates and automated counts of live motor neurons, defined as GFP^+^ neurons with significant neurite outgrowth (SNO, total neurite length >75 µm), were performed (Figures 1A and 1B) [31]–[33]. In standard culture medium without neurotrophic support motor neuron numbers decreased over the first 7 days, reaching a plateau that was maintained until day 31+13 (Figure 1C and 1D). This did not reflect a loss of reporter expression since a similar decrease was seen when motor neurons were identified by staining for endogenous HB9 (not shown). In contrast, when the medium was supplemented with four neurotrophic factors [NTFs; brain-derived neurotrophic factor (BDNF), ciliary neurotrophic factor (CNTF), glial cell line-derived neurotrophic factor (GDNF) and insulin-like growth factor 1 (IGF-1) at 10 ng/mL] in addition to the cAMP-elevating compounds forskolin (F; 10 µM) and isobutylmethylxanthine (I; 100 µM), after an initial decrease in motor neuron numbers by day 31+7, there was a subsequent increase in the number of hESC-MNs, which reached nearly starting levels by day 31+13 (Figure 1C and 1D).

This late increase in human motor neuron numbers could potentially be explained by ongoing genesis of motor neurons. To assess overall generation of new-born motor neurons we cultured cells with or without NTFs in the continuous presence of the mitotic label 5’-bromo-2’deoxyuridine (BrdU, 2 µM) and counted GFP-positive cells that had incorporated BrdU (Figure 1E). After 15 days, ∼60% of all Hb9::GFP cells were positive for BrdU in both conditions (Figures 1F and 1G), but cultures supplemented with NTFs contained 4-fold higher absolute numbers of new-born hESC-MNs (Figure 1G, p<0.05). Together, these results demonstrate that human motor neurons are generated over extended periods of culture and that the yield of motor neurons can be increased by treatment with neurotrophic factors.

### Screening for small molecules able to increase the yield of human motor neurons

Neurotrophic factors are costly culture supplements and have pleiotropic effects on neural development [16], [34], [35]. To exploit the high rate of neurogenesis in hESC-MN cultures in a more targeted manner to increase motor neuron yields, we sought to identify available reagents with similar activity. We therefore performed a small-scale screen of 160 bioactive compounds selected from a collection of drug-like chemicals and examined their effect on total motor neuron numbers. We reasoned that the assay might capture two types of compounds, those that increase motor neuron survival and/or others that increase motor neurogenesis. Both types of compounds would be of interest as they could be applied to increase the overall yields of motor neurons derived from hESCs.

**Figure 2:**
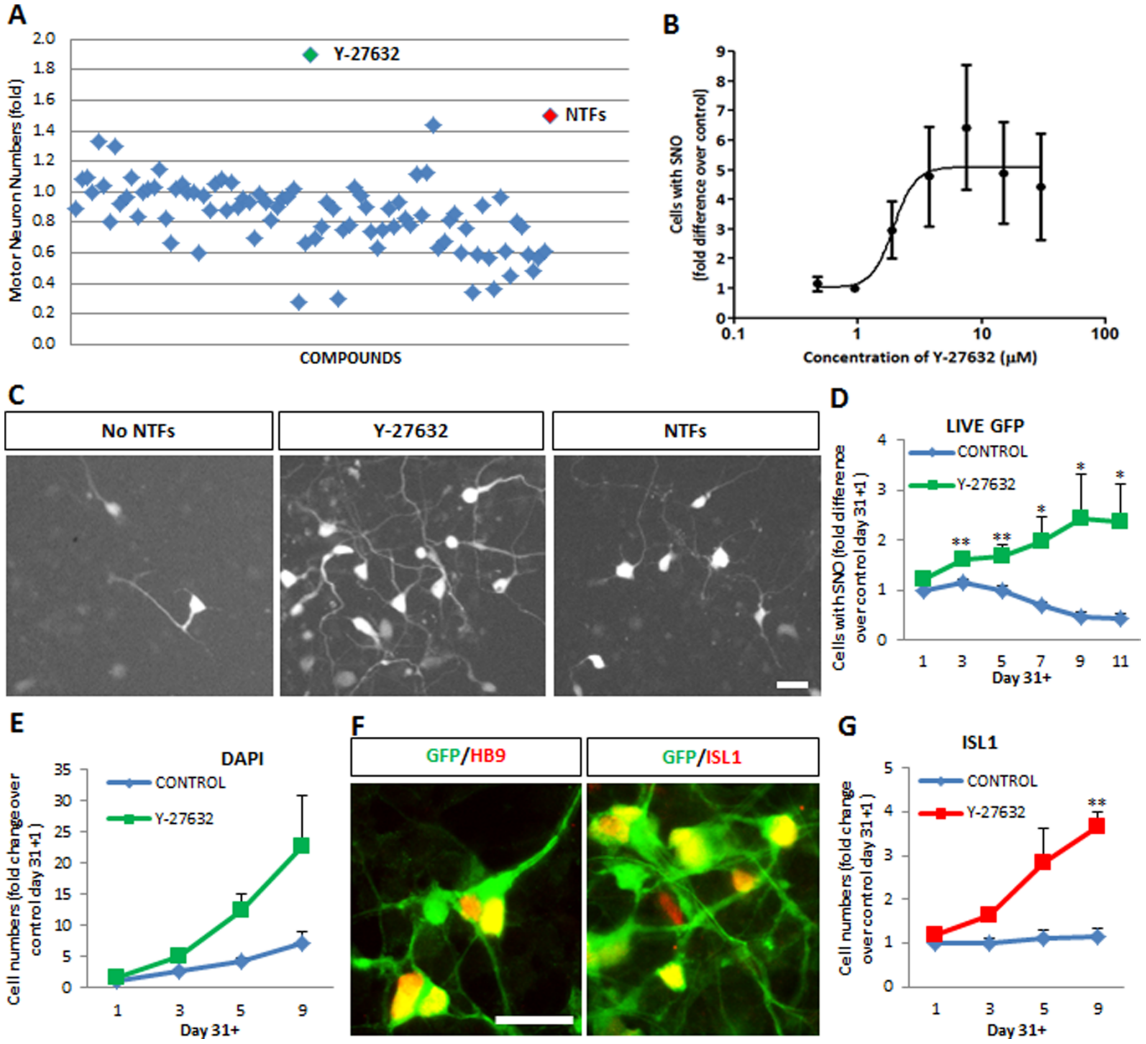
The ROCK inhibitor Y-27632 increases human motor neuron numbers in hESC-derived motor neuron cultures. (A) Screening of 160 compounds for their potential to increase the number of human motor neurons in hESC cultures at day 31+13. Compounds were tested in quadruplicate at a single concentration (10 µM). Values are plotted as mean fold difference in motor neuron numbers relative to the negative control condition (No NTFs). The Rho-kinase (ROCK) inhibitor Y-27632 was the compound showing the highest capacity to increase the number of human motor neurons. (B) Y-27632 increases the number of fluorescent hESC-motor neurons in mixed cultures in a dose-dependent manner. Cells were cultured in the absence of neurotrophic factors and in the presence of increasing concentrations of Y-27632. Values shown as mean ± s.e.m., *n* = 4. (C) Representative images of hESC-motor neuron cultures at day 31+13 grown under neurotrophic factor deprivation (No NTFs), neurotrophic factor supplementation (NTFs + F + I) and Y-27632 (10 µM). Scale bar = 25 µM. (D) Time-dependent increase in the number of motor neurons in the presence (green) but not absence (blue) of Y-27632 (10 µM), with a peak effect at day 31+9. Values shown as mean ± s.e.m., *n*>5 (t-test, *p<0.05; **p<0.01). (E) Y-27632 also increases the total number of cells in culture. Mean ± s.e.m., *n* = 3. (F) Hb9::GFP-positive neurons continue to express motor neuron markers HB9 and ISL1 after treatment with Y-27632 for 9 days. Scale bar = 50 µM. (G) Supplementation of cultures with Y-27632 (red line) leads to increased numbers of human motor neurons expressing endogenous ISL1 at day 31+9. Mean ± s.e.m., *n* = 3 (**p<0.01).

Compounds (10 µM in quadruplicate wells) were added on the day of seeding and motor neurons were counted at day 31+13, the time point at which the greatest differences in human motor neuron numbers between control and NTF-supplemented cultures were observed (Figure 1C, p<0.001). Most compounds showed no effect, and a significant number resulted in lower motor neuron numbers than the negative control condition (Figure 2A). In contrast, two compounds increased motor neuron numbers by>1.4 fold compared to basal conditions (Figure 2A). The most significant increase (1.9-fold) was induced by the Rho kinase (ROCK) inhibitor Y-27632 (Figure 2A, Y-27632 vs. No NTFs, p<0.05).

The EC_50_ for Y-27632 at day 31+13 was 1.9 µM with a maximum effect of ∼5-fold (Figure 2B; p<0.05), even greater than that of neurotrophic factors (Figures 2A and 2C; Y-27632 vs. NTFs + F + I, p<0.05). To optimize the time window for the effects of Y-27632, we next studied the kinetics of human motor neuron generation with or without Y-27632 at its optimal concentration (10 µM). We focused on its effects when added post-dissociation at day 31. Maximum numbers of GFP-positive neurons, representing a ∼5-fold increase in motor neuron numbers over basal levels (Figure 2D, p<0.05) were reached at day 31+9, which was therefore adopted as the standard time point for all subsequent experiments.

To exclude the possibility that Y-27632 might have affected the fidelity of HB9 reporter expression, we checked day 31+9 cultures using direct immunostaining for the motor neuron markers ISL1 and HB9; both showed a high degree of overlap with the GFP reporter (Figure 2F). Moreover, Y-27632 induced a nearly 4-fold increase in absolute numbers of hESC-MNs expressing endogenous ISL1 (Figure 2G, p<0.05). Furthermore, to exclude the possibility that the class of motor neurons generated was altered with respect to standard differentiation protocols, we quantified the fraction of GFP-positive neurons expressing FoxP1, a marker for limb-innervating motor neurons, or Lhx3, a marker of medial motor neurons [30], [36], [37]. Comparable numbers of each class were generated and the ratio was not significantly affected by amplification with Y-27632 (p>0.05; not shown). One potential risk of this amplification procedure was that Y-27632 might dilute out motor neurons by stimulating the generation of other cell types. However, this did not appear to negatively affect the outcome: across the many different batches of hES-MNs analyzed in this study, the final abundance of motor neurons ranged from 5% to 45% of total cells, making it important that all treatment groups be compared to controls from the same batch. Although we did not exclude the batches with lower abundance, the value of 45% motor neurons is among the highest reported, demonstrating that expansion did not lead to excessive motor neuron depletion. Thus using a small-scale drug testing approach we were able to identify a compound, Y-27632, which can significantly increase motor neuron numbers in differentiated hESC cultures.

### Y-27632 enhances proliferation of motor neuron progenitors in both hESC- and hiPSC-derived cultures

**Figure 3:**
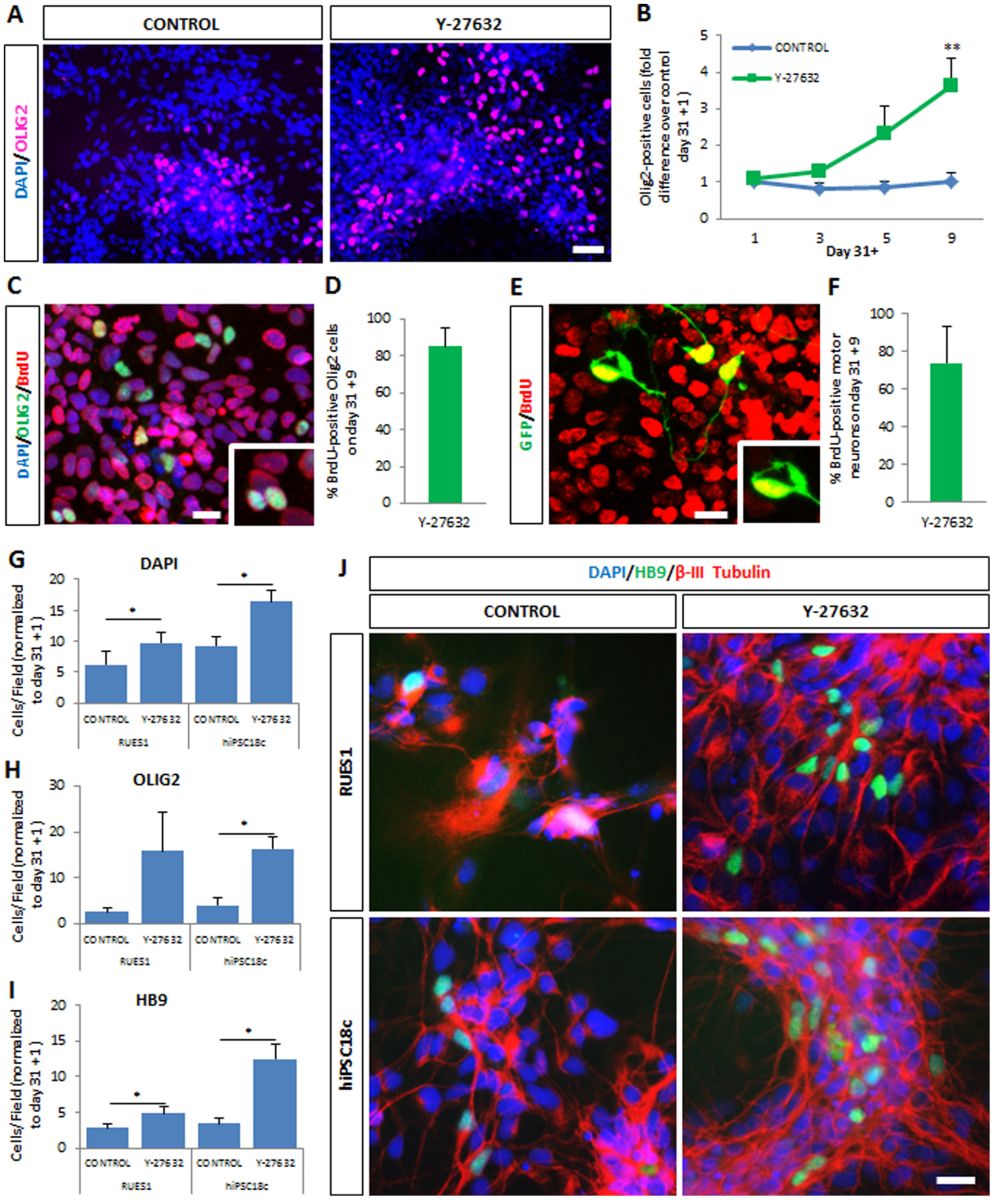
Y-27632 enhances proliferation of motor neuron progenitors in hESC- and hiPSC-derived motor neuron cultures. (A) Y-27632-supplemented cultures contain increased numbers of OLIG2-positive cells at day 31+9. Scale bar = 50 µM. (B) Time-dependent increase in numbers of OLIG2-expressing progenitors in the presence of Y-27632. Data normalized to control at day 31+1; mean ± s.e.m., *n*>5 (t-test, **p<0.01). (C) OLIG2 progenitors at day 31+9 stained for BrdU. Scale bar = 25 µM. (D) Percent of OLIG2 precursors that are BrdU-positive at day 31+9 (mean ± s.e.m., *n* = 4). (E) Hb9::GFP-expressing motor neurons at day 31+9 stained for BrdU. Scale bar = 25 µM. (F) Percent motor neurons that are BrdU-positive at day 31+9 (mean ± s.e.m., *n* = 4). (G) The total number of cells in culture is increased at day 31+9 following Y-27632 treatment of hESC RUES1 and hiPSC18c. Values are mean ± s.e.m., *n*≥3 (t-test, *p<0.05). (H) Numbers of OLIG2 precursors increase significantly at day 31+9 following Y-27632 treatment of hiPSC 18c. Values are mean ± s.e.m., *n*≥3 (t-test, *p<0.05). (I) Numbers of motor neurons identified by staining for endogenous HB9 increase significantly at day 31+9 following Y-27632 treatment of hESC RUES1 and hiPSC 18c. Values are mean ± s.e.m., *n*≥3 (t-test, *p<0.05). (J) Cultures from healthy control hESCs (RUES1) or hiPSCs (18c) immunostained for the motor neuron marker HB9 and the pan-neuronal marker β-III tubulin. Y-27632 increases the number of motor neurons in each case. Scale bar  =  25 µM.

To better understand the level at which Y-27632 exerts its effect, we next examined the expansion of motor neuron progenitors (pMNs), using OLIG2 as a marker [26]. Treatment with Y-27632 increased the number of OLIG2-positive cells ∼3.6-fold compared to controls by day 31+9 (Figures 3A and 3B, p<0.05) comparable to the ∼3.3-fold increase in DAPI-stained cells over controls over the same period [Figure 2E, p>0.05; Ratio DAPI/OLIG2 = 6.8∶1 (CONTROL) vs. 6.2∶1 (Y-27632)]. Application of BrdU from day 31 to day 31+9 led to nuclear labeling of 86% of OLIG2-positive cells, indicating that they are actively proliferating progenitors (Figures 3C and 3D). Accordingly, 74% of GFP-positive motor neurons on day 31+9 were BrdU-positive, indicating that they were born during the period of Y-27632 treatment (Figures 3E and 3F). Similar percentages were observed using fresh, unfrozen motor neuron preparations (not shown). Therefore Y-27632 non-selectively enhances cell proliferation in hESC-derived cultures, resulting in a ∼3.5-fold increase in the number of motor neuron progenitors that is likely to contribute significantly to the observed increase in postmitotic hESC-MNs.

To determine whether Y-27632 was a generally effective treatment for pluripotent stem cell lines, we performed similar experiments using an additional hESC line, RUES1; and a hiPSC line, 18c, derived from a healthy control subject [6]. Total numbers of DAPI-stained cells and OLIG2-positive progenitors were quantified as above after 31+9 days. Significant increases in both DAPI-positive and OLIG2-positive cells were observed following Y-27632 treatment using hiPSC 18c (Figures 3G and 3H, p<0.05) and for DAPI using RUES1 (Figure 3G). To detect motor neurons in the absence of a reporter we performed immunostaining for HB9, to label motor neurons, and β-III tubulin, to label all neurons (Figures 3I and 3J). Automated image analysis of such cultures revealed a 2- to 4-fold increase in motor neuron numbers (Figure 3I, p<0.05). Y-27632 is therefore a useful tool for both hESCs and clinically relevant hiPSC lines.

### Design of a robust survival assay for purified human motor neurons

**Figure 4:**
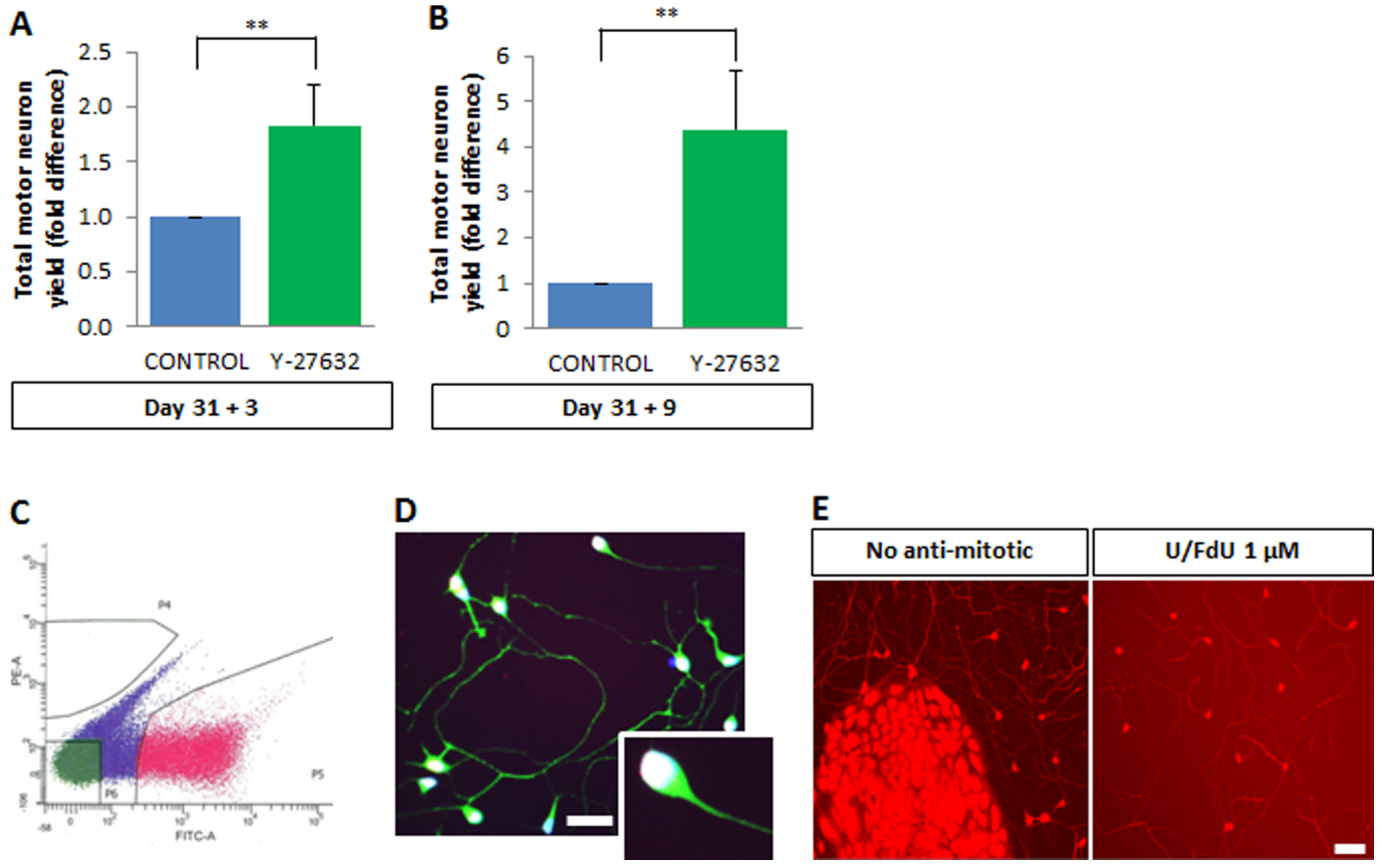
FACS-sorting of amplified cultures yields a pure preparation of viable human motor neurons. (A) Y-27632 supplementation for 3 days leads to a 1.8-fold increase in motor neuron yield judged by FACS analysis. Data normalized to controls without Y-27632. Values are mean ± s.e.m., n>5 (t-test, **p<0.01). (B) Nine-day treatment with Y-27632 gives a ∼5-fold increase in motor neuron yield as compared to controls without Y-27632, as quantified by flow cytometry. Values are mean ± s.e.m., n>5 (t-test, **p<0.01). (C) FACS purification of Hb9::GFP motor neurons expanded with Y-27632 for 3 days. Representative FACS gating used to retrieve an almost pure (>95%) population of human motor neurons. (D) FACS-purified motor neurons at day 31+3+1 stained for GFP (green), and a combination of HB9 and ISL1 (“pan-MN”; white nuclei).>95% of the FACS-purified cells in culture are Hb9::GFP positive. Scale bar  =  25 µM. (E) Even following FACS sorting, some contaminant cells were able to proliferate and form colonies that interfered with survival assays (left panel). Uridine/Fluorodeoxyuridine (U/FdU) (each at 1 µM) successfully prevented the proliferation (right panel).

Our overall goal was to study the trophic requirements of human motor neurons. Bulk day 31 cultures were therefore dissociated and grown in the presence of Y-27632 for 3 days or 9 days before FACS analysis, leading to a ∼2-fold increase in the total yield of motor neurons after 3 days (Figure 4A; p<0.01) and a nearly 4-fold increase after 9 days (Figure 4B; p<0.01). For all subsequent experiments, expanded human motor neurons from the day 31+3 time point were used.

Given the ongoing neurogenesis in mixed cultures, it was first necessary to find conditions in which expanded postmitotic neurons could be studied in isolation. Direct treatment of mixed cultures with mitotic inhibitors did not produce satisfactory results: cytosine arabinoside (AraC) proved toxic for human motor neurons, while even the less toxic uridine/fluorodeoxyuridine (U/FdU) led to clumping of neurons on remaining islands of non-neuronal cells (not shown). Motor neurons were therefore FACS-sorted (Figure 4C) and seeded on polyornithine/laminin-coated coverslips in medium containing a cocktail of NTFs plus the c-AMP elevating compounds forskolin and IBMX. Using FACS conditions involving a slow sorting rate and a wide nozzle, the seeded motor neurons rapidly developed robust neurite outgrowth (Figure 4D). To estimate their purity, we performed immunostaining using a combination of antibodies to HB9 and ISL1 (“pan-MN”) [30]. At day 31+3+1 (differentiation + expansion + days post-FACS),>95% of the neurons were Hb9::GFP-positive, and reporter expression showed strong overlap with HB9/ISL1 staining (Figure 4D). Despite this high degree of enrichment, colonies of proliferating progenitors were occasionally observed (Figure 4E); sorted motor neurons were therefore cultured in the presence of the antimitotic drug U/FdU (Figure 4E). The new protocol therefore provides a robust and abundant source of highly purified hESC-MNs.

**Figure 5:**
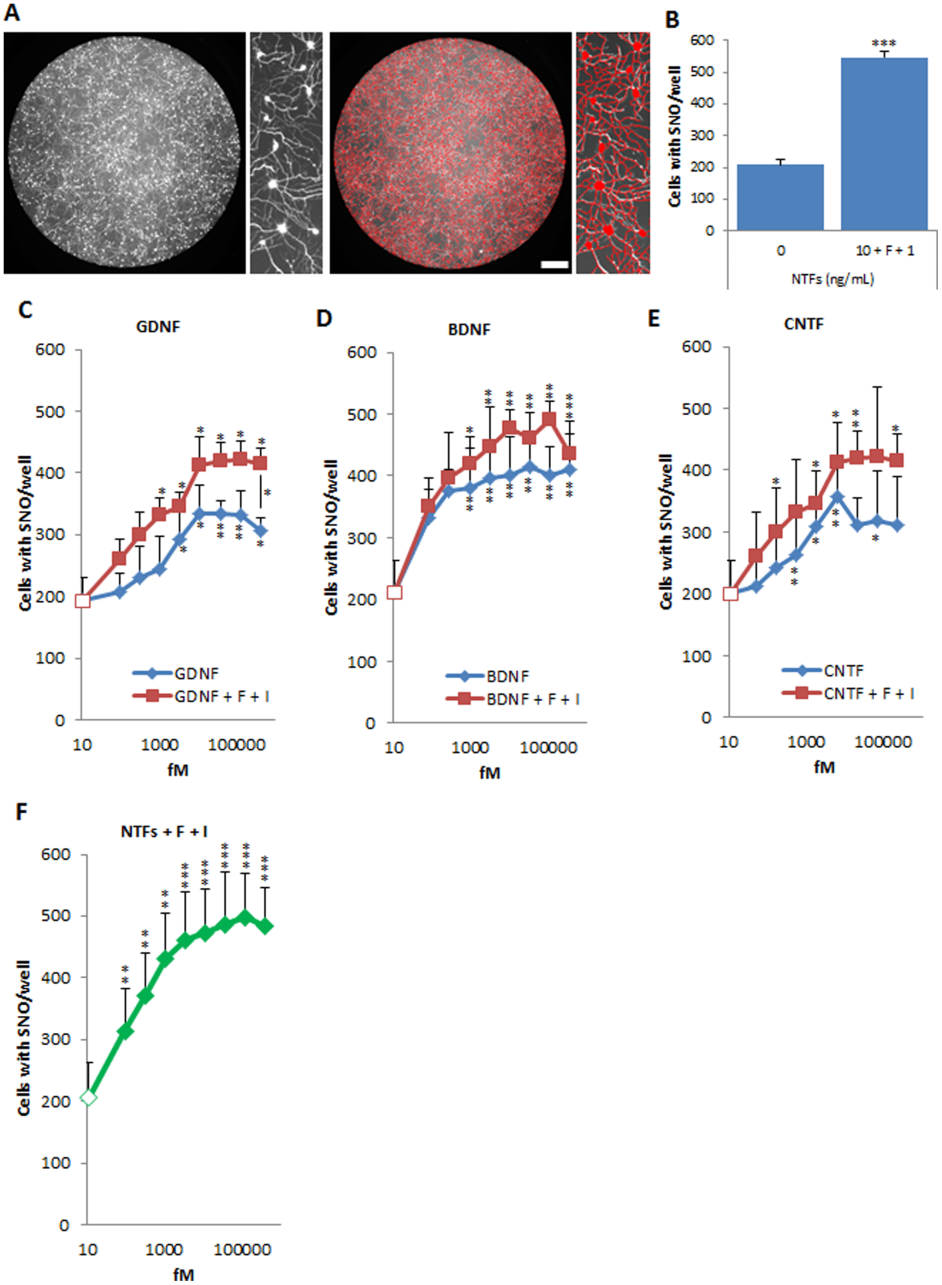
Purified human motor neurons show a potent response to known neurotrophic factors. (A) Whole-well imaging of live motor neurons labeled with calcein-AM captured using the Plate Runner (left two panels). Surviving human motor neurons were counted in whole culture wells in an automated manner using MetaMorph (red tracing, right two panels). Scale bar = 200 µm. (B) Y-27632-expanded motor neurons show enhanced survival in the presence of a cocktail of neurotrophic factors. Values shown as mean ± s.e.m., n>5 (t-test, ***p<0.001). (C) GDNF, (D) BDNF and (E) CNTF alone (blue lines) enhance the survival of expanded FACS-purified human motor neurons. The addition of F+I significantly potentiates the survival-inducing activity of GDNF at high concentrations. Values shown as mean ± s.e.m., n>4 (t-test, *p<0.05; **p<0.01; ***p<0.001). Asterisks on individual points represent significance of difference with No-NTF control (white rectangle in the curve); asterisks on bars represent significant differences between a given concentration of NTF and the corresponding value for NTF + F+ I. (F) The cocktail of neurotrophic factors (NTFs) enhances the survival of expanded FACS-purified human motor neurons in a dose-dependent manner in the presence of 10 µM forskolin plus 100 µM IBMX. Values shown as mean ± s.e.m., n≥5 (t-test, **p<0.01; ***p<0.001).

To develop a survival assay based on neurotrophic factor deprivation [31], [38], [39], FACS-sorted motor neurons were seeded in 96-well plates and stained using the vital dye calcein-AM. This had the advantage that it stained cell bodies and neurites more intensely than live imaging of GFP, which was no longer required to identify motor neurons. Numbers of surviving hESC-MNs were counted in whole culture wells in an automated manner using MetaMorph (Figure 5A). We first asked whether the survival of purified motor neurons was dependent on trophic support in these conditions. At day 31+3+7, motor neuron survival was enhanced ∼2.5-fold by a cocktail of NTFs (BDNF, CNTF, GDNF, IGF-1, each at 10 ng/ml) with F (10 µM) plus IBMX (100 µM) (Figure 5B), similar to published results using cultures of primary rodent motor neurons [39]–[41]. We tested forskolin and IBMX alone and found that they showed only slight innate neurotrophic activity (not shown).

This provided an opportunity to better characterize the effects of known neurotrophic factors on hESC-MNs. Doses of GDNF, BDNF, CNTF and IGF-1 ranging from 2 pg/mL to 10 ng/mL were first tested alone for their effects on survival at day 31+3+7 (Figure 5D to 5G). Except for IGF-1 (not shown), which showed no survival promoting effect alone, each neurotrophic factor provided significant support for human motor neuron survival with EC_50_ values as follows: 2 pM for BDNF, 2 pM for GDNF and 1 pM for CNTF. These are slightly higher than the most potent EC_50_ values reported for the same factors on primary rodent motor neurons (BDNF, EC_50_ = 1 pM [38]; GDNF, EC_50_ = 0.2 pM [31]; CNTF, EC_50_ = 0.1 pM [18], [42]); this may reflect differences related to species, human stem cell origin or batch of neurotrophic factor. Since the effects of neurotrophic factors on rat motor neurons in defined media was reported to depend on intracellular cAMP levels [40], [41], we also tested the effects of inclusion of forskolin and IBMX (F+I). The neurotrophic activity of each factor tested appeared to be increased in the presence of F+I, though this effect was only significant for single points at the highest concentration of GDNF (Figure 5D-F). To determine whether different neurotrophic factors were potentially acting on different subsets of motor neurons in the cultures, we next performed a dose-response analysis for a combination of all factors with a fixed concentration of F+I (Figure 5H). The maximum number of motor neurons maintained in culture was not significantly greater than that with BDNF, CNTF or GDNF alone (with F+I). This suggests that essentially all viable motor neurons are maintained by optimal doses of these single factors, at least after 7 days in culture. Therefore, like their rodent counterparts, human motor neurons show an exquisitely sensitive response to multiple neurotrophic factors.

**Figure 6:**
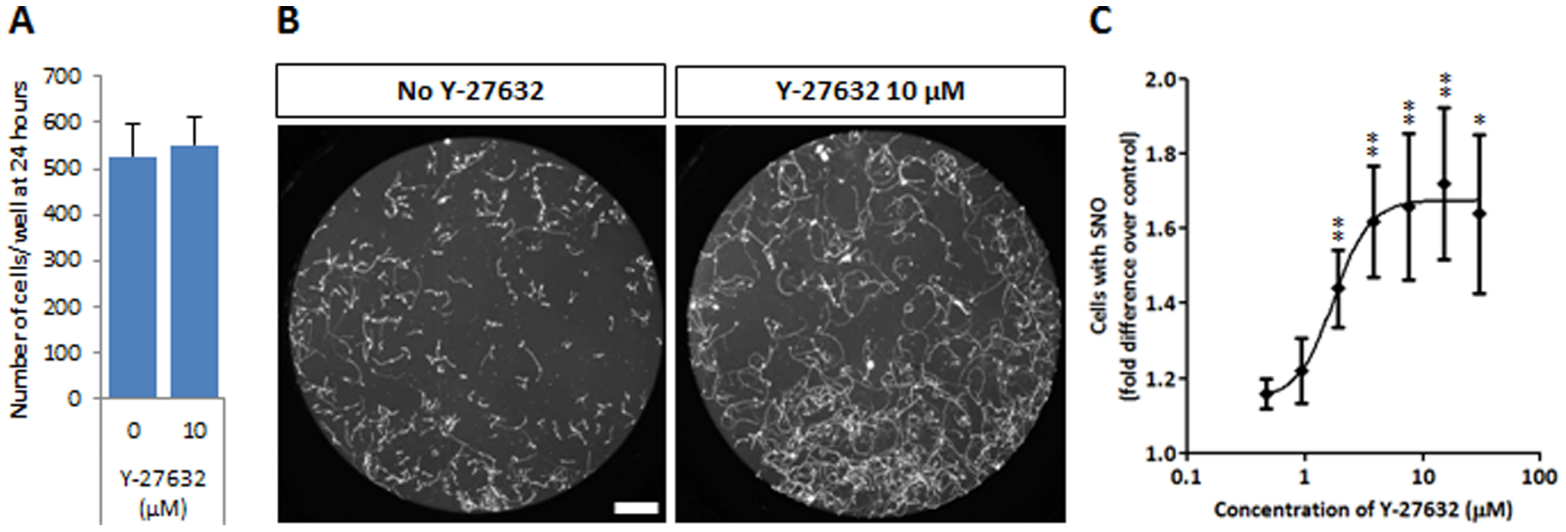
Y-27632 is also a survival factor for human motor neurons. (A) The plating efficiency of FACS-purified human motor neurons after 24 hours is not increased in the presence of Y-27632. (B) Y-27632 enhances the survival of FACS-purified human motor neurons in a 7-day survival assay. Scale bar = 200 µM. (C) Dose-dependent effects of Y-27632 on human motor neuron survival, expressed relative to the basal condition (0 µM). Values shown as mean ± s.e.m., n≥5 (t-test, *p<0.05; **p<0.01).

Lastly, to evaluate the ability of the newly developed human motor neuron survival assay to detect novel neurotrophic compounds, we determined whether the beneficial effect of Y-27632 on human motor neuron numbers, in addition to its effect on progenitor proliferation, might also reflect a survival effect. To exclude effects on cell attachment we first verified that the presence of the drug did not affect hESC-MN numbers after 24 hours (Figure 6A). After 7 days in culture, Y-27632 had a clear dose-dependent survival effect (Figures 6B and 6C), though to a more modest extent than neurotrophic factors. The EC_50_ for the Y-27632 survival effect on motor neurons was 2 µM, similar to the value for motor neuron expansion. Thus Y-27632 not only promotes proliferation of motor neuron progenitors but also functions as a motor neuron survival factor. The fact that the fold-increase in survival was lower than that induced in long-term treatment of mixed cultures (Fig. 2B), likely reflects the absence of proliferation and/or other cell types.

## Discussion

Human embryonic and induced pluripotent stem cells (hESCs and hiPSCs) represent a powerful tool for studying human development, disease modeling and drug discovery. However, one major limiting factor for prospective drug screens is the efficiency with which the affected cell types can be generated and, in the case of motor neurons and many other neuronal classes, the absence of a validated survival assay. Here, we took advantage of our observation of ongoing motor neuron generation in hESC-derived cultures to devise a new method for amplification of motor neuron progenitors to increase motor neuron yields. In addition, using optimized conditions for FACS sorting of neurons expressing the Hb9::GFP reporter, we developed a robust assay for survival factors acting directly on postmitotic motor neurons, and used it to show that human motor neurons respond in a potent manner to both known and novel neurotrophic molecules.

The ongoing neurogenesis in human motor neuron cultures that we describe contrasts with the short ∼24-hour period of motor neuron production in differentiated mouse ES cell cultures [43]. This is likely to reflect normal biological differences in the development of motor systems in rodent and human embryos, since human motor neurons are produced over an extended three-week period *in vivo* [28], [29]. We first exploited this to screen for compounds that would further amplify the precursor population, identifying Y-27632 as the most active compound in a screen which, like higher-throughput assays, was carried out at a single concentration. Exactly how Y-27632 is achieving this may involve multiple mechanisms, but we considered three potential modes of action for Y-27632 in increasing numbers of motor neuron progenitors. First, it could act by blocking differentiation of progenitors to motor neurons. This seems unlikely since the numbers of OLIG2-positive precursors and motor neurons increased in parallel. Second, it might specifically promote the generation of OLIG2-positive precursors. Since total DAPI numbers increased in parallel, such a selective effect seems unlikely. We therefore believe Y-27632 acts by shortening cell cycle time for dividing precursors as a whole, leading to expansion – but not enrichment – of motor neuron progenitors and a subsequent increase in motor neuron yield [44]–[46].

Nevertheless, the ongoing neurogenesis also provides a potentially serious confound for interpretation of experiments examining changes in motor neuron numbers in mixed cultures. In studies that do not take this into account, it is possible that an increase of motor neuron numbers attributed to improved survival may instead reflect an effect on neurogenesis. To overcome this issue, we FACS-purified motor neurons derived from the Hb9::GFP hESC line and cultured them alone in the presence of a mitotic inhibitor U/FdU to inhibit proliferation of any remaining progenitors. This is in some ways analogous to the approach recently reported by Yang et al. [24], except that to block proliferation they used cytosine arabinoside, which was cytotoxic in our hands. Moreover, their cell survival experiments, performed over a 20-day period, required a mouse astrocyte monolayer as substrate, whereas our cultures contained essentially only motor neurons.

Using this essentially pure preparation of postmitotic motor neurons we showed that three known neurotrophic factors potently enhance human motor neuron survival, and that their action is potentiated when endogenous levels of cAMP are increased. Therefore, in this respect, the human stem cell-derived neurons closely resemble rodent motor neurons both in primary culture and *in vivo*. Since dependence on trophic factors is acquired over time during embryogenesis [47], this also suggests that the human motor neurons have reached a stage of maturation comparable to those in the mid-embryonic period in mice.

Y-27632 has been shown to have contrasting biological effects in different systems, ranging from pro-proliferative effects on hESCs and hiPSCs [48], [49] to anti-proliferative effects on cancer cells [50], cord blood-derived CD34^+^ hematopoietic progenitor cells [51], hepatic stellate cells [52] and smooth muscle cells [53]. While it is neuroprotective for primary mouse Purkinje cells [54], retinal ganglion cells [55] and murine hippocampal slice cultures [56] and growth-promoting for corticospinal tract axons [57], [58] and adult optic nerve [59], Y-27632 is not protective for hiPSC-derived dopaminergic neurons [60]. Our study extends others which suggest that Y-27632 exhibits generally beneficial effects on motor neurons. A recent report documented an increase in the lifespan of an intermediate mouse model of SMA following administration of fasudil, another ROCK inhibitor [61]. Even though the compound was not able to halt motor neuron loss in the ventral horn of the spinal cord, positive effects on the maturation of the neuromuscular junction and muscle fiber size were reported [61]. More recently, fasudil was reported to extend survival – and reduce motor neuron death - in a mouse model of amyotrophic lateral sclerosis [62]. Therefore, the neurotrophic properties of Y-27632 described here for cultured human neurons likely reflect a mechanism of action that is conserved across species and *in vivo*.

In summary, our study defines conditions for systematic assays of neurotrophic factors and survival-promoting compounds for human motor neurons. We show that the technique can be extended to human iPSC-derived motor neurons and therefore in principle to comparisons between cells derived from ALS patients and controls: we and others recently derived Hb9::GFP or Hb9::RFP reporters for different ALS-iPSC lines [63]. Importantly, in agreement with our earlier studies on the expression of specific markers, electrophysiological characteristics and development following transplantation [6], [30], we show that the neurotrophic dependence of human stem cell-derived motor neurons has reached a state of maturity comparable to that of primary embryonic motor neurons *in vitro* and *in vivo*. Although more still needs to be done before they can be considered to reflect the properties of the postnatal spinal cord, this validates their use as a human model for analyzing multiple aspects of motor neuron development and pathology.

## Materials and Methods

### Cell lines

All the human ES and iPSC lines have been reported in an earlier publication [6]. The iPS cell lines were derived by retroviral transduction of OCT4, SOX2, and KLF4 in dermal fibroblasts. All pluripotent cell lines were characterized by conventional methods and grown under standardized conditions as described below.

### Ethics Statement

The work performed with human motor neurons derived from hESCs and hiPSCs has been approved by Columbia University ESCRO committee (Embryonic Stem Cell Research Oversight committee). Patient fibroblasts for generating human iPS lines were collected with written informed consent under IRB approval AAAC1257 from Columbia University Medical Center.

### Growth of hPSC lines

We used an HB9::GFP reporter hESC line [23], the wild-type hESCs line RUES1 and hiPSC line 18c [6]. All cell cultures were maintained in a humidified incubator at 37°C and 5% CO_2_. Human ESCs and hiPSCs were grown on a pre-gelatinized tissue culture flask on a monolayer of irradiated CF-1 mouse embryonic fibroblasts (MEFs; GlobalStem) plated at 15,000–18,000 cells/cm^2^ in hPSC medium [DMEM/F12 (Invitrogen), 20% knockout serum replacement (Invitrogen), 1 mM L-glutamine (Gibco), 100 µM non-essential aminoacids (Gibco) and 100 µM β-mercaptoethanol (Sigma-Aldrich)] supplemented with 20 ng/ml recombinant human basic fibroblast growth factor (bFGF; R&D Systems). Medium was changed every day for the duration of the expansion and lines were passaged every 4–6 days using dispase (Gibco) at 1 mg/mL in hPSC medium for 30 minutes at 37°C.

### Differentiation of hESCs and hiPSCs into motor neurons

hESCs and hiPSCs were allowed to reach 75%–90% confluency. Then, colonies were treated with dispase (1 mg/mL) to separate colonies from the MEF layer. After 30 minutes, cells were washed off the flask using hPSC medium and collected in a 50 mL Falcon tube. Colonies were allowed to settle by gravity and then medium was aspirated. Fresh hPSC medium was added to the cells. This step was repeated three times to wash away all the remaining dispase. Settled colonies were then mechanically dissociated into small 10- to 15-cell chunks using a P1000 tip by performing up and down movements in a 1 mL volume. Cell aggregates were transferred to low adherence T75 flasks in hPSC medium with 20 ng/mL bFGF and 20 µM Y-27632 (Ascent) for the first 24 hours. At day 1, cells for all experiments were supplemented with hPSC medium containing 20 ng/mL bFGF, 20 µM Y-27632, 10 µM SB431542 (Sigma-Aldrich) and 0.2 µM LDN193189 (Stemgent). The medium was changed daily from day 2 to day 4. At day 5, embryoid bodies (EBs) were switched to medium composed of 50% hPSC medium and 50% neural induction medium [NIM; DMEM/F12 (Invitrogen), 1% N2 supplement (Invitrogen), 1 mM L-glutamine (Gibco), 100 µM non-essential aminoacids (Gibco) and 2 µg/mL heparin (Sigma-Aldrich)] supplemented with 10 µM SB431542, 0.2 µM LDN193189, 10 ng/mL recombinant human brain-derived neurotrophic factor (BDNF; R & D Systems), 0.4 µg/mL ascorbic acid (Sigma-Aldrich) and 1 µM retinoic acid (Sigma-Aldrich). At Day 7 cells were switched to 100% NIM, keeping the same medium supplementation. Every other day between days 9 and 21, NIM supplemented with 10 ng/mL BDNF, 0.4 µg/mL ascorbic acid, 1 µM retinoic acid and 200 ng/mL recombinant C25II modified sonic hedgehog protein (SHH; Invitrogen) was added to the EBs. At day 22, cells were cultured with 50% NIM and 50% neural differentiation medium [NDM; Neurobasal (Invitrogen), 1% N2 Supplement (Invitrogen), 1 mM L-Glutamine (Gibco) and 100 µM Non-Essential Aminoacids (Gibco)] supplemented with 2% B-27 supplement (Invitrogen), 0.4 µg/mL ascorbic acid, 1 µM retinoic acid, 200 ng/mL SHH (Invitrogen), 10 ng/mL BDNF, 10 ng/mL recombinant human ciliary neurotrophic factor (CNTF; R & D Systems), 10 ng/mL recombinant human glial cell line-derived neurotrophic factor (GDNF; R & D Systems) and 10 ng/mL recombinant human insulin-like growth factor 1 (IGF-1; R & D Systems). Between days 24 and 31, the bulk medium was switched to 100% NDM and the EBs grown under the previous medium supplementation. After 31 days of differentiation the EBs were dissociated and the resulting neuronal cultures cryopreserved. Briefly, the EBs were collected in a 50 mL Falcon tube and then washed twice with PBS without Ca^2+^ and Mg^2+^ (Invitrogen) to eliminate residual media. The EBs were then incubated at 37°C in pre-warmed 0.05% Trypsin-EDTA (Invitrogen) for 5–10 minutes. Lastly, fetal bovine serum (Invitrogen) supplemented with 100 µg/mL deoxyribonuclease I (DNAse I, Sigma-Aldrich) was added to stop the trypsin reaction and the cells were spun for 5 minutes at 400×*g*. The cells were resuspended in 1 mL of complete trituration and wash medium [CTWM, PBS without Ca^2+^ and Mg^2+^, 25 mM Glucose (Sigma-Aldrich), 4% L-15 dialyzed BSA (Sigma-Aldrich), 100 µg/mL DNAse I, 1% N2 supplement, 2% B27 supplement, 600 mM magnesium chloride (Sigma-Aldrich), 500 nM EDTA (Sigma-Aldrich) and 2% FBS] and subsequently mechanically triturated using a P1000 tip. The resulting cell suspension was filtered using a 40 µM cell strainer (BD Falcon) to eliminate large residual clumps and centrifuged for 5 minutes at 400×*g*. The cells were then resuspended in NDM supplemented with 2% B27, 0.4 µg/mL ascorbic acid, 25 µM glutamate E (Sigma-Aldrich), 25 µM β-mercaptoethanol (Millipore), 0.1 µM retinoic acid, 10 ng/mL BDNF, 10 ng/mL CNTF, 10 ng/mL GDNF and 10 ng/mL IGF-1. These cells were counted and then prepared for cryopreservation using 2x Freezing Media (Millipore). Vials of 5–10 million cells/mL were prepared to be used in further experiments.

### Coating of 96-well plates

All survival and proliferation studies were performed in 96-well plates (Greiner Bio-One) coated with polyornithine (Sigma-Aldrich) and mouse laminin (Invitrogen). Briefly, 100 µg/mL polyornithine (Sigma-Aldrich) in cell culture water was added to the wells for at least 2 hours then aspirated and the wells rinsed once using water. Coating was completed by adding overnight 15 µg/mL mouse laminin in L15 medium (Sigma-Aldrich) supplemented with 7.5% sodium bicarbonate (Gibco). In studies involving FACS-sorted cells, a concentration of 1000 µg/mL polyornithine was used for coating.

### Studies involving mixed hPSC-derived motor neuron cultures

All proliferation/survival studies involving mixed hPSC-derived motor neuron cultures were started from previously cryopreserved vials. After quickly thawing the vials in a 37°C water bath, cells were resuspended in NDM medium supplemented with 2% B27 Supplement. Cells were then spun at 400×*g* for 5 minutes. The supernatant was gently aspirated and cells resuspended in 10 mL of NDM with 2% B27. A 4% BSA protein cushion was then layered under the cell suspension and the cells spun at 400×*g* for 5 minutes, with low acceleration and deceleration. Afterwards, cells were resuspended in basal medium (BM) [Custom Clear Neurobasal (Invitrogen), which omits phenol red and riboflavin to allow live fluorescent imaging in the presence of a significantly attenuated auto-fluorescent background; 1 mM L-glutamine and 100 µM non-essential aminoacids, 2% B27, 0.4 µg/mL ascorbic acid, 25 µM glutamate E, 25 µM β-mercaptoethanol, 0.1 µM retinoic acid] and counted using a hemocytometer. Finally, cells were resuspended at the final desired seeding concentration of 32,000 cells/well and 100 µL was added to each well. Cells were allowed to attach at 37°C for 2 hours before addition of supplements at 3x concentration in 50 µL of BM.

### Screening for small molecules with the potential to increase numbers of human motor neurons in culture

From a collection of drug-like chemicals from the Microsource and Tocris collections, two plates containing a total of 160 compounds were selected. Each compound was tested at 10 µM. Basal medium to dilute compounds from original stocks was M-199 (without phenol red; Invitrogen) with 5% DMSO (100% anhydrous, Fisher Scientific), freshly prepared. Survival in BM was used as negative control (trophic factor deprivation). Survival in BM supplemented with a cocktail of NTFs [BDNF, CNTF, GDNF and IGF-1] plus the cAMP-elevating compounds forskolin (F; 10 µM; Sigma-Aldrich) and isobutylmethylxanthine (I; 100 µM; Sigma-Aldrich) was the positive control (trophic factor supplementation). Cells were seeded at 32,000 cells/well in 150 µL and compounds added in a 15 µL volume (the final DMSO concentration of 0.45% did not adversely affect motor neuron survival when added alone, not shown). The same volume of M-199 with 5% DMSO was added to the negative control wells. NTFs + F + I were also added in 15 µL of M-199 with 5% DMSO in positive control wells. For each set, compounds were tested in quadruplicate by creation of 4 test plates. In each plate, each control condition (positive and negative) had six replicate wells. Readouts were performed on day 31+13. After quantification of the total number of surviving cells with significant neurite outgrowth (see *Results*), data were plotted as mean fold difference as compared to numbers in the negative control condition. Plates were rejected when the mean difference in cells numbers between positive and negative control was lower than 1.3 fold. Validation of the most active compounds was performed by serial dose response studies.

### Immunocytochemistry

Neuronal cultures were pre-fixed by adding one volume of 4% paraformaldehyde diluted in phosphate-buffered saline 1x (PBS1x/4%PFA) for 2 minutes at room temperature. Then, cells were fixed with PBS1x/4%PFA for 30 minutes at 4°C. After fixation, cells were washed with PBS1x three times for 5 minutes and then permeabilized and quenched for at least 30 minutes using PBS1x with 0.1% Triton-X (PBSTX-0.1%) supplemented with 100 mM glycine and 0.1% Sodium Azide (Sigma-Aldrich). Cells were blocked in PBSTX-0.1% containing 10% donkey serum (Sigma-Aldrich) and 0.1% sodium azide (Sigma-Aldrich) (blocking solution) for one hour. After blocking, cells were incubated overnight at 4°C with primary antibodies diluted in the blocking solution. Primary antibodies used in this study were the following: rabbit anti-GFP (1∶3000, Abcam), mouse anti-ISL1 (1∶200, DSHB, 39.4D5), guinea-pig anti-ISL1 (1∶2000, courtesy of Susan Brenner-Morton, Jessell laboratory at Columbia University), mouse anti-HB9 (1∶100, DSHB, MNR2 81.5C10-c), chicken anti-β-III Tubulin (TUJ1, 1∶1000, Neuromics), rabbit anti-Olig2 (1∶1000, Millipore) and rat anti-BrdU (1∶150, Serotec). Cells were washed five times with PBSTX-0.1% for 5 minutes. Antigens were visualized by incubating for 60–75 minutes at room temperature with the appropriate secondary antibodies (DyLight 488, 549 and 649 conjugated, 1∶1000, Jackson ImmunoResearch). Lastly, neuronal cultures were again washed five times with PBSTX-0.1% for 5 minutes and incubated in a solution containing DAPI (1∶50000, Sigma-Aldrich) for 15 minutes. Cells were washed once with PBSTX-0.1% and then imaged.

### BrdU incorporation studies

5-bromo-2-deoxuridine (BrdU) incorporation studies were performed to analyze cell proliferation. Neuronal cultures were incubated with BrdU (2 µM; Sigma-Aldrich) for the full duration of culture until fixation with PFA. The standard protocol for immunochemistry described above was followed for other antigens besides BrdU. Then, to detect BrdU incorporation, cells were again pre-fixed for 2 minutes at room temperature and fixed with PBS1x/4% PFA for 15 minutes at 4°C. They were then washed with PBS1x three times for 5 minutes. Cells were then incubated with pre-warmed (37°C) 2 M HCl in distilled water for 10 minutes at 37°C, light protected. Lastly, the HCl was aspirated and cells were incubated in 0.15 M boric acid in distilled water for 2 minutes at room temperature. Cells were then washed three times with PBS1x for 5 minutes and blocked for 1 hour using blocking solution. Finally, cells were incubated overnight at 4°C with rat anti-BrdU primary antibody (1∶150, Serotec) in blocking solution. In order to correct for any non-specific background staining, the same procedures were performed on samples incubated or not with BrdU.

### FACS purification and motor neuron survival studies

HB9::GFP reporter hESC-derived motor neurons were grown in polyornithine/laminin-coated T75 flasks prior to FACS purification in order to maximize the amount of cells retrieved after the procedure. After the expansion period, the medium was aspirated and cells washed once with PBS without Ca^2+^ and Mg^2+^ to eliminate residual medium. The cells were then incubated at 37°C in pre-warmed 0.05% Trypsin-EDTA for 5 minutes. DNAse I-supplemented FBS was used to stop the trypsin reaction. Cells were collected and centrifuged for 5 minutes at 400×*g*. Cells were resuspended in complete trituration and wash medium [CTWM, PBS without Ca^2+^ and Mg^2+^, 25 mM Glucose (Sigma-Aldrich), 4% L-15 dialyzed BSA (Sigma-Aldrich), 100 µg/mL DNAse I, 1% N2 supplement, 2% B27 supplement, 600 mM magnesium chloride (Sigma-Aldrich), 500 nM EDTA (Sigma-Aldrich) and 2% FBS], filtered through a 40 µM Cell Strainer (BD Falcon) and centrifuged for 5 minutes, at 400×*g*. The cells were then resuspended in 750–800 µL of CTWM and transferred to a sorting tube (BD Falcon). Cells were sorted based on GFP expression using a BD FACS Aria II sorter (Becton Dickinson) configured with a 100 µm ceramic nozzle and operating at 20 psi for no longer than 30 minutes. Purified cells were collected in a tube containing CTWM. After collection cells were spun for 5 minutes at 400*xg* and resuspended in Basal FACS Medium [Basal Medium with Clear Custom Neurobasal, supplemented with 1 µM uridine/fluorodeoxyuridine (U/FdU; Sigma-Aldrich), 100 Units/mL Penicillin (Invitrogen), 100 µg/mL Streptomycin (Invitrogen) and 100 µg/mL Normocyn (InvivoGen)]. After cells were counted using a hemocytometer, they were resuspended at the final seeding concentration of 2000 cells/well and added to the wells in 100 µL. Medium supplements were added to the cells at 3x concentration in 50 µL of Basal FACS Medium after the cells were allowed to incubate at 37°C for 2 hours in order to attach to the bottom of the plate. Readouts were performed after 7 days.

### Calcein live imaging

To facilitate the imaging of FACS-sorted GFP-positive cells, we used the Calcein-AM Red-Orange (Invitrogen) vital dye at 2.5 µM concentration. Briefly, cells were incubated with the dye for 5 minutes and then extraneous fluorescence was quenched using 5 mg/mL hemoglobin before image acquisition using the Plate Runner (Trophos).

### Image acquisition and quantitative image analysis

Image acquisition was performed using either a Carl Zeiss Observer Z1 epi-fluorescence Microscope (Carl Zeiss Inc.; acquisition of 12 images per well at 10x magnification) or the whole well imaging device Plate Runner (Trophos). Automated quantitative image analysis of fluorescent surviving hESC-MNs and stained neuronal cultures was performed using the MetaMorph Software V7.6 (Molecular Devices). The Neurite Outgrowth application in the software was employed to quantify fluorescent human motor neurons that have neurite outgrowth above a certain threshold, reducing the number of false-positive cells such as non-viable neurons included in the analysis. Quantitative analysis of stained hPSC-derived human motor neuron cultures was performed using the Multi-Wavelength Cell Scoring application. For a specific marker, positive cells were selectively identified as having clear signal intensity above local background. Intensity thresholds were set blinded to sample identity. In a given experiment the same parameters were used in all images analyzed. Parameters were only minimally adjusted across different experiments.

### Statistical Analyses

All quantitative data were analyzed using IBM SPSS Statistics 19 (IBM SPSS). For each set of data a double statistical evaluation was performed: A) for each condition/time point mean values were compared using one-way ANOVA statistical evaluation followed by Tukey HSD Post-hoc test; B) possible interactions between time and condition were assessed using two-way ANOVA statistical evaluation. In cases involving only one time point and a two-group comparison, p value was determined using Student's t-test. Differences were considered to be significant when p<0.05.
