# Correction: Neurotrophic Requirements of Human Motor Neurons Defined Using Amplified and Purified Stem Cell-Derived Cultures

**DOI:** 10.1371/journal.pone.0119195

**Published:** 2015-03-18

**Authors:** The PLOS ONE Staff

There is an error in the second sentence of the fourth paragraph in the “Design of a robust survival assay for purified human motor neurons” section of the Results. The correct sentence is: Doses of GDNF, BDNF, CNTF and IGF-1 ranging from 2 pg/mL to 10 ng/mL were first tested alone for their effects on survival at day 31+3+7 (Figure 5C to 5F).

The incorrect scale bar was used in the figure legends for Figs. 1, 2, 3, 4 and 6. The scale bar should be μm instead of μM. The authors have provided corrected versions of Figs. 1, 2, 3, 4, and 6 here.

**Fig 1:**
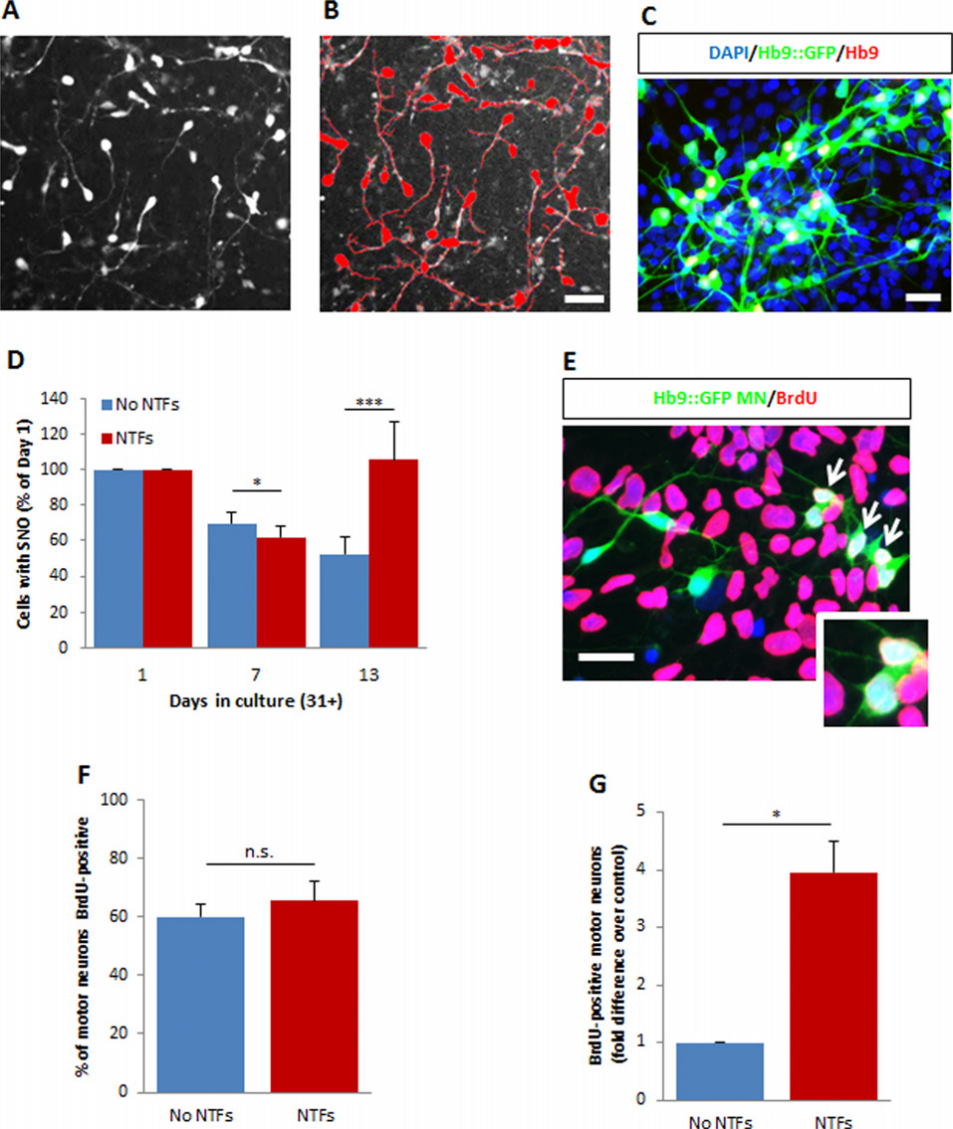
Ongoing birth of motor neurons in hESC-derived cultures is stimulated by neurotrophic factors. (A) Live fluorescent human motor neurons derived from the Hb9::GFP reporter line at day 31+13 after growth with a cocktail of neurotrophic factors (NTFs). (B) Automated quantification of fluorescent cells with significant neurite outgrowth (SNO) using the Neurite Outgrowth module of MetaMorph software; cells counted are identified with a red overlay. Motor neurons were considered to have significant neurite outgrowth when their overall neurite length exceeded 75 μm (scale bar). (C) Representative image of immunostained Hb9::GFP hESC-motor neuron cultures at day 31+13 after growth with a cocktail of neurotrophic factors (NTFs). Scale bar = 50 μm. (D) Number of cells with significant neurite outgrowth (SNO) when grown with (red bars) or without (blue bars) neurotrophic factors, expressed as a percentage of numbers at day 31+1. The increase in motor neuron numbers after day 31+7 in NTF-supplemented cultures suggests ongoing neurogenesis. Surviving fluorescent GFP-positive motor neurons with SNO shown as mean ± s.e.m., n>5 (t-test, ***p<0.001, *p<0.05). (E) BrdU-positive Hb9::GFP-positive motor neurons (arrows) at day 31+15 confirming the presence of newborn human motor neurons in culture. Scale bar = 50 μm. (F) The percentage of Hb9::GFP-positive motor neurons that were BrdU-positive at day 31+15 is not changed by NTFs but (G) total numbers of BrdU-positive motor neurons are increased with NTFs. Bars indicate mean ± s.e.m., n = 3 (t-test, *p<0.05; n.s. = not significant).

**Fig 2:**
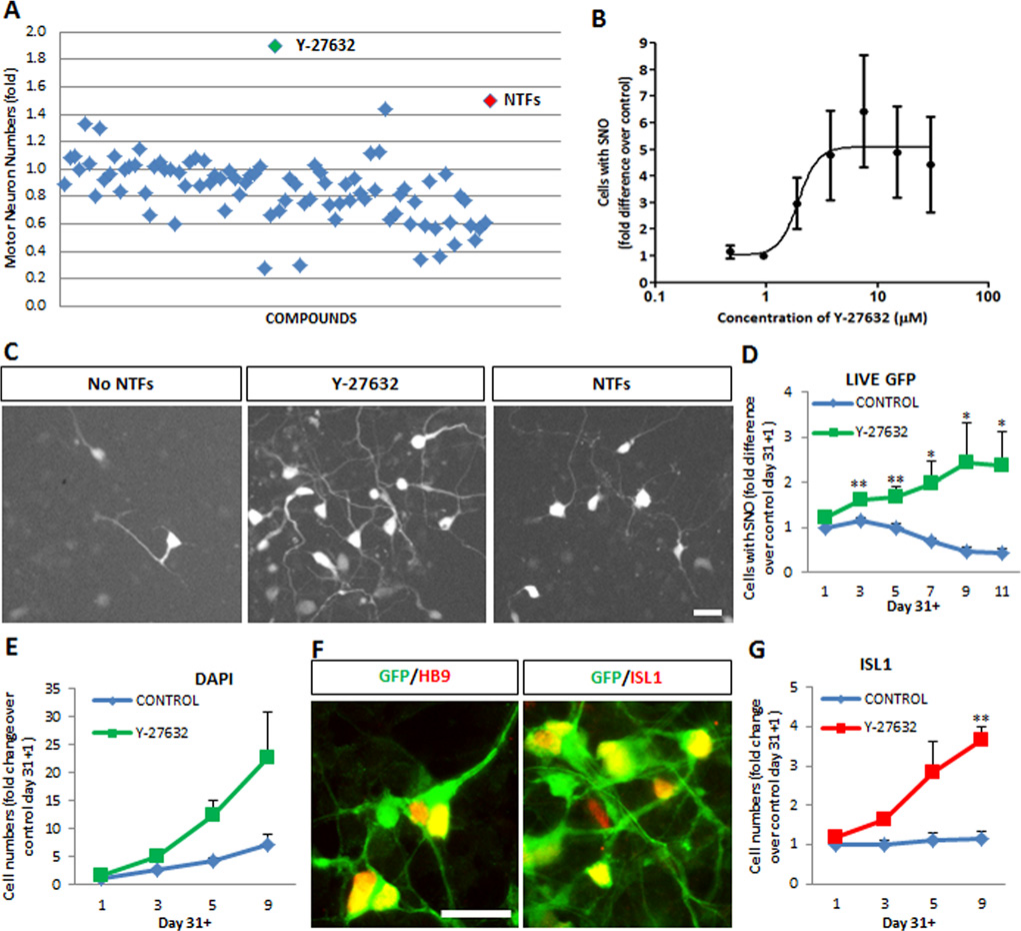
The ROCK inhibitor Y-27632 increases human motor neuron numbers in hESC-derived motor neuron cultures. (A) Screening of 160 compounds for their potential to increase the number of human motor neurons in hESC cultures at day 31+13. Compounds were tested in quadruplicate at a single concentration (10 μM). Values are plotted as mean fold difference in motor neuron numbers relative to the negative control condition (No NTFs). The Rho-kinase (ROCK) inhibitor Y-27632 was the compound showing the highest capacity to increase the number of human motor neurons. (B) Y-27632 increases the number of fluorescent hESC-motor neurons in mixed cultures in a dose-dependent manner. Cells were cultured in the absence of neurotrophic factors and in the presence of increasing concentrations of Y-27632. Values shown as mean ± s.e.m., n = 4. (C) Representative images of hESC-motor neuron cultures at day 31+13 grown under neurotrophic factor deprivation (No NTFs), neurotrophic factor supplementation (NTFs + F + I) and Y-27632 (10 μM). Scale bar = 25 μm. (D) Time-dependent increase in the number of motor neurons in the presence (green) but not absence (blue) of Y-27632 (10 μM), with a peak effect at day 31+9. Values shown as mean ± s.e.m., n>5 (t-test, *p<0.05; **p<0.01). (E) Y-27632 also increases the total number of cells in culture. Mean ± s.e.m., n = 3. (F) Hb9::GFP-positive neurons continue to express motor neuron markers HB9 and ISL1 after treatment with Y-27632 for 9 days. Scale bar = 50 μm. (G) Supplementation of cultures with Y-27632 (red line) leads to increased numbers of human motor neurons expressing endogenous ISL1 at day 31+9. Mean ± s.e.m., n = 3 (**p<0.01).

**Fig 3:**
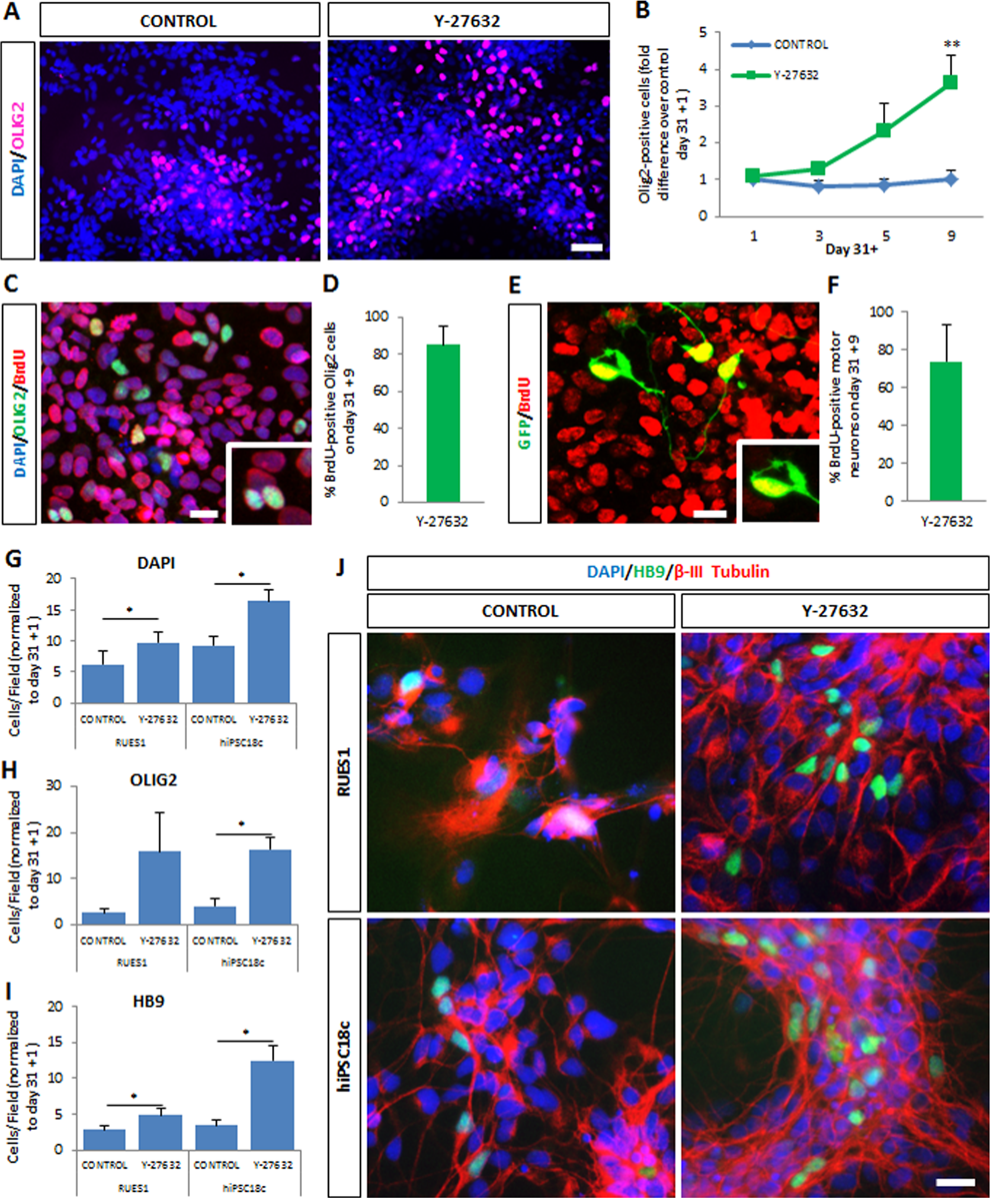
Y-27632 enhances proliferation of motor neuron progenitors in hESC- and hiPSC-derived motor neuron cultures. (A) Y-27632-supplemented cultures contain increased numbers of OLIG2-positive cells at day 31+9. Scale bar = 50 μm. (B) Time-dependent increase in numbers of OLIG2-expressing progenitors in the presence of Y-27632. Data normalized to control at day 31+1; mean ± s.e.m., n>5 (t-test, **p<0.01). (C) OLIG2 progenitors at day 31+9 stained for BrdU. Scale bar = 25 μm. (D) Percent of OLIG2 precursors that are BrdU-positive at day 31+9 (mean ± s.e.m., n = 4). (E) Hb9::GFP-expressing motor neurons at day 31+9 stained for BrdU. Scale bar = 25 μm. (F) Percent motor neurons that are BrdU-positive at day 31+9 (mean ± s.e.m., n = 4). (G) The total number of cells in culture is increased at day 31+9 following Y-27632 treatment of hESC RUES1 and hiPSC18c. Values are mean ± s.e.m., n≥3 (t-test, *p<0.05). (H) Numbers of OLIG2 precursors increase significantly at day 31+9 following Y-27632 treatment of hiPSC 18c. Values are mean ± s.e.m., n≥3 (t-test, *p<0.05). (I) Numbers of motor neurons identified by staining for endogenous HB9 increase significantly at day 31+9 following Y-27632 treatment of hESC RUES1 and hiPSC 18c. Values are mean ± s.e.m., n≥3 (t-test, *p<0.05). (J) Cultures from healthy control hESCs (RUES1) or hiPSCs (18c) immunostained for the motor neuron marker HB9 and the pan-neuronal marker β-III tubulin. Y-27632 increases the number of motor neurons in each case. Scale bar = 25 μm.

**Fig 4:**
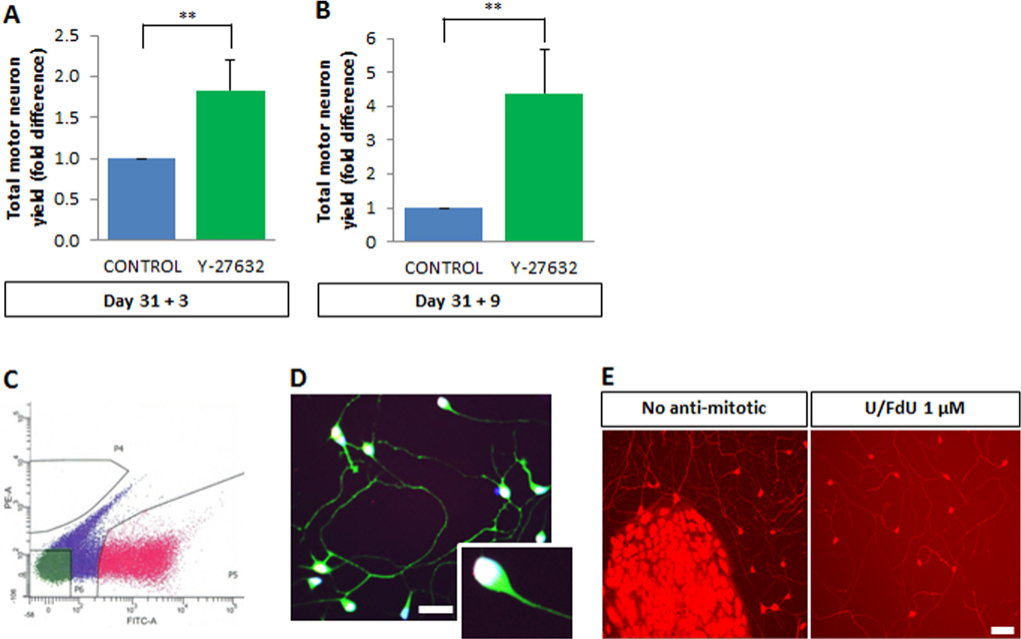
FACS-sorting of amplified cultures yields a pure preparation of viable human motor neurons. (A) Y-27632 supplementation for 3 days leads to a 1.8-fold increase in motor neuron yield judged by FACS analysis. Data normalized to controls without Y-27632. Values are mean ± s.e.m., n>5 (t-test, **p<0.01). (B) Nine-day treatment with Y-27632 gives a ~5-fold increase in motor neuron yield as compared to controls without Y-27632, as quantified by flow cytometry. Values are mean ± s.e.m., n>5 (t-test, **p<0.01). (C) FACS purification of Hb9::GFP motor neurons expanded with Y-27632 for 3 days. Representative FACS gating used to retrieve an almost pure (>95%) population of human motor neurons. (D) FACS-purified motor neurons at day 31+3+1 stained for GFP (green), and a combination of HB9 and ISL1 (“pan-MN”; white nuclei).>95% of the FACS-purified cells in culture are Hb9::GFP positive. Scale bar = 25 μm. (E) Even following FACS sorting, some contaminant cells were able to proliferate and form colonies that interfered with survival assays (left panel). Uridine/Fluorodeoxyuridine (U/FdU) (each at 1 μM) successfully prevented the proliferation (right panel).

**Fig 6:**
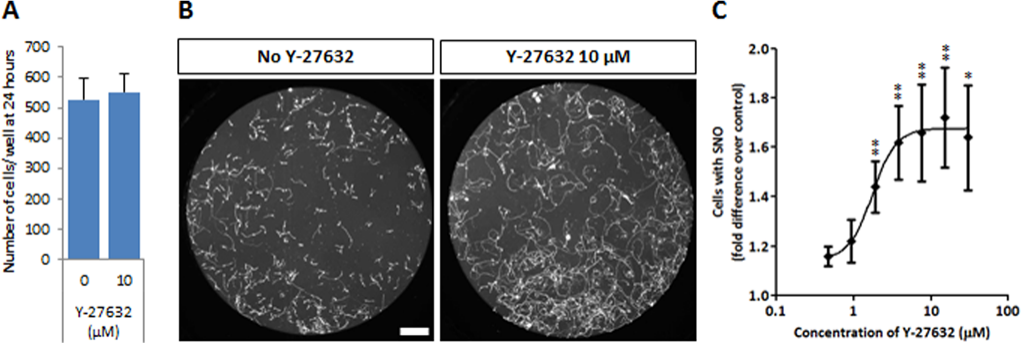
Y-27632 is also a survival factor for human motor neurons. (A) The plating efficiency of FACS-purified human motor neurons after 24 hours is not increased in the presence of Y-27632. (B) Y-27632 enhances the survival of FACS-purified human motor neurons in a 7-day survival assay. Scale bar = 200 μm. (C) Dose-dependent effects of Y-27632 on human motor neuron survival, expressed relative to the basal condition (0 μM). Values shown as mean ± s.e.m., n≥5 (t-test, *p<0.05; **p<0.01).
